# Extraction of* Amana edulis* Induces Liver Cancer Apoptosis

**DOI:** 10.1155/2018/3927075

**Published:** 2018-07-04

**Authors:** Yueyang Fan, Xiaowei Hou, Pengpeng Guo, Xiuhua Lv, Lingling Zhao, Huilan Wang, Lu Zhou, Yinglu Feng

**Affiliations:** ^1^Department of Traditional Chinese Medicine, PLA 401 Hospital, Qingdao Shandong, China; ^2^No. 2 High School of East China Normal University, China; ^3^Department of Oncology, PLA 401 Hospital, Qingdao Shandong, China; ^4^Institute of Integrative Medicine, Qingdao University Medical College, Qingdao Shandong, China; ^5^Department of Medicinal Chemistry, School of Pharmacy, Fudan University, 826 Zhang-Heng Rd., Shanghai 201203, China

## Abstract

HCC is one of the fastest-rising causes of cancer-related death. Novel therapeutic approaches for treatment are warranted. The goal of this study is to find effective components from Chinese herbal medicines, which is an important alternative source of anticancer medicine. To this end, six different herbs were selected from various traditional literatures. Soxhlet extractor was used to distill the strong polar and weak polar components of each herb. The inhibitive effect of each component was determined using liver cancer cells BEL7404. From total of 12 extractions, it was found that the combined crude lysate of* Amana edulis* from water and ethanol system had the best efficacy. At the concentration of 0.1 mg/mL, this component has the highest inhibition rate up to 70%. To investigate the underlying molecular reasons, we observed that the component can significantly induce the liver cancer cells apoptosis and retard the cell reproduction at G2/M stage. Verification experiments showed that this component also has apparent inhibitive effects on other liver cancer cells, such as HepG2 and Huh7. On the other hand, it has less effectiveness on another cell line HepaRG, which retains many characteristics of primary human hepatocytes. The results suggested that there might be highly efficient antihepatoma ingredient in the water and ethanol extraction of* Amana edulis*. The pure substances remain to be isolated and further research on their targets is required.

## 1. Introduction

Hepatocellular carcinoma (HCC) has become one of the leading causes of death and an important public health problem in China because of the increasingly higher morbidity and mortality rates. It was reported that the death number from HCC ranks second in China, while the current treatment and drug R&D remain backward [1]. Many HCC were detected at advanced stage due to its extended initiation and progression times, with poor liver function, high tumor recurrence rate, and metastasis. Unfortunately, most advanced HCC are unresectable [2]. A variety of therapies have been used for the treatment of liver cancer, such as chemotherapy, radiotherapy, cryo-ablation, and transcatheter arterial chemoembolization (TACE) [3]. However, the effects of these treatments are poor due to unresponsiveness of HCC cells and are often accompanied by multiple side effects including bone marrow depression, hair loss, postembolization syndrome, and liver and renal failure.

Traditional Chinese medicine (TCM) is a medical science studying disease prevention, diagnosis and treatment, rehabilitation, and health preservation. It has been used in China for thousands of years [4–6]. It has made a significant contribution to the treatment of various diseases [7]. Herbal medicine is one of the major components in TCM. It was originated from the first literature of* Shennong's Materia Medica* (~220 CE). Herbal medicine is an important treasure of Chinese nation, which has been used for thousands of years and gained remarkable effects on the treatment of various diseases including tumor, due to accumulating abundant theoretical and practical knowledge through thousands of years [8], and the abundant herb resource has also provided essential material foundation to look for new anticancer drugs [9–11]. The modern success of using taxol and artemisinin further proves its value. A plenty of Chinese herbs medicines have been proved effective in treating liver diseases. Research results also showed that some Chinese herbal medicines are commonly used for patients with HCC in the clinical practice of Chinese medicine practitioners [12]. HCC is deadly and has become a public health problem. However, most antitumor drugs in clinical cases, including molecular targeted drug and antibody drug, are Western medicine, only a few native compounds or their derivatives with significant effect. Thus, in the present study, we got the idea for research on fighting against cancer with Chinese medicine. We plan to look for less-focused Chinese medicinal herbs with potential antitumor activity by consulting classic ancient literatures about Chinese herbal medicine for separation and purification of active components to research their antitumor activity, anticipating to find new natural products with anticancer activity.

Although there are few records of such disease names of “tumor” or “cancer” in traditional medical works, the description of occurrence, development, diagnosis, and treatment to many diseases, such as “Yingliu” (goiter), “Luoli” (scrofula), “Zhengjiajiju”(gynecologic abdominal agglomerate), and “Yeshi” (oesophageal carcinoma), is of high similarity to “tumor” and “cancer” in modern medicine. Hereby, in the present study, by consulting a massive of ancient literatures, including “Treatise on Febrile Diseases (Shanghan Lun)”, “Huangdi's Canon of Medicine”, and “Compendium of Materia Medica”, we selected these medicinal herbs of* Amana edulis, Prunella vulgaris L.*,* Vaccaria segetalis* (*Neck*.)* Garcke*, Gnaphalium* affine D. Don, Paeonia lactiflora Pall., and Bupleurum chinense DC *by taking the words of “Jiju, Jiejie, and Zhengjia” as the basis and under the principle of “no repetitive, effectiveness, hypotoxicity, and nonscarcity”.

We identified that the combined crude lysate of* Amana edulis* from water and ethanol system had the highest inhibition efficacy. Our results further showed that crude lysate of* Amana edulis *could significantly induce the liver cancer cells into early and late stage apoptosis and retard the cell reproduction at G2/M stage. These data suggested that there may be highly efficient antihepatoma component in* Amana edulis*.

## 2. Materials and Methods

### 2.1. Experimental Materials and Reagents

*Amana edulis*,* Prunella vulgaris L.*,* Vaccaria segetalis (Neck.) Garcke*,* Gnaphalium affine D. Don*,* Paeonia lactiflora Pall.*, and* Bupleurum chinense DC* were all purchased from Tongrentang Pharmacy and kept in an airtight device for a period of time before processing.

### 2.2. Cell Culture

The human hepatoma cell lines of BEL7404 were cultured in Roswell Park Memorial Institute (RPMI) 1640 Medium supplemented with 10% fetal bovine serum (FBS) [13, 14] and incubated under the condition of 5% CO2 humidified atmosphere at 37°C with saturated humidity [15, 16].

### 2.3. Soxhlet Extraction Method

100g of Chinese herbal medicines was extracted using ethanol-water solvent mixed system (100mL of water and 100mL of ethyl alcohol) and petroleum ether-ethyl acetate mixed system (100mL of petroleum ether and 100mL of ethyl acetate) for 120min by heat reflux extraction, respectively. Then solvents were removed with vacuum rotary evaporation. The extractions were kept at −20°C until used.

### 2.4. Cell Viability Assay

Cell Counting Kit-8 was used to evaluate the cytotoxicity of* Amana edulis*, as described in a previous paper [17]. Cells were seeded in 96-well plates at 20,000 cells per well and added various concentrations of* Amana edulis* and incubated for 24h. CCK-8 solution was added to each well and the plate is placed in the incubator for 4h after which the absorbance was read at 450nm using VERSAmax tunable microplate reader (Sunnyvale, CA, USA).

### 2.5. MTT Assay

In order to further verify the cell proliferation of crude lysate of* Amana edulis*, 3-(4,5-dimethylthiazol-2-yl)-2,3-diphenyl tetrazolium bromide (MTT) assay was conducted in accordance with a previously described method [18] in the four cell lines of BEL7404, HepG2, Huh7, and HepaRG cells. Briefly, 1×10^4^ cells in 100 *μ*L medium were plated into each well of 96-well plates (five wells per group). 24h after plating, 10*μ*L 0.5mg/mL MTT solution was added to each culture well, and then the cells were incubated at 37°C for 4h. Then, the MTT solution in each culture well was removed. Finally, 150*μ*L of DMSO was added to each culture well, and the mixtures were shaken. The absorbance was measured on a microplate reader (Multiskan Spectrum; Thermo Fisher, Waltham, MA, USA) at 540nm.

### 2.6. Annexin V-FITC Assay

Cell apoptosis was analyzed using the Annexin V-FITC/PI Apoptosis Detection Kit according to the manufacturer's protocol (Sigma-Aldrich Co., St. Louis, MO) with slight modifications. Briefly, BEL7404 cells were plated in 96-well plates at a density of 4×10^5^ cells/mL. After 24-hour incubation, cells were treated with crude lysate of* Amana edulis* and then incubated at 37°C for 24h. Cells were harvested by trypsinization and centrifugation, and cell supernatant was abandoned. The cells were washed three times with the precold PBS buffer, and the cells density was adjusted to 1×10^6^ cell/mL. Then Annexin V and PI solution were added to the cells at room temperature without light exposure for 15min. Cell apoptosis was determined using flow cytometer (EasyCyte guava, Merck Millipore).

### 2.7. Cell Cycle Analysis

Cells were seeded in 96-well plates at a density of 4×10^5^ cells/mL and incubated at 37°C to allow cells to grow to 60%–80% confluence. Cells were treated with crude lysate of* Amana edulis* for 24h. Cells were collected by digestion and fixed with 70% ethanol at 4°C overnight. Then the cells were resuspended in phosphate-buffered saline (PBS) and incubated at 37°C for 1h. Finally, the cells were stained with propidium iodide (PI) for 30min without light exposure. Stained cells were analyzed by flow cytometer (EasyCyte guava, Merck Millipore). ModFit Cell Cycle Analysis software was used to analyze the percentage of cells in G1, G2, and S phases based on DNA content.

### 2.8. Statistical Analyses

The experiments were repeated three times, and the data represent the means. The results were analyzed using one-way ANOVA followed by Student's t-test [19, 20]. An inspection level was considered to indicate a significant difference when p < 0.05 [21, 22].

## 3. Results

### 3.1. The Inhibition Rate of Six Ingredients

**Figure 1:**
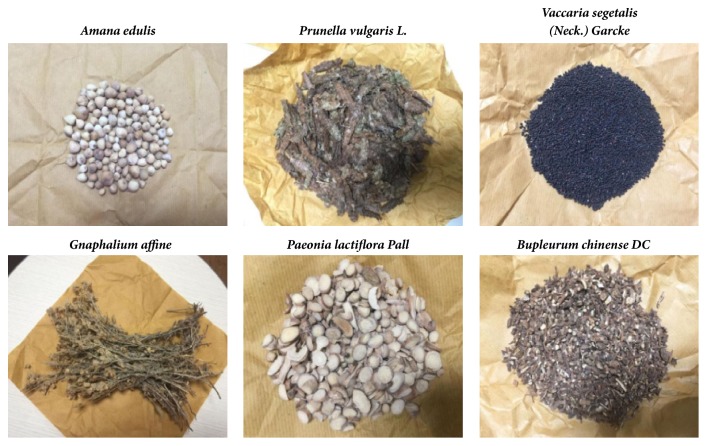
Images of six commonly used Chinese herbal medicines.

We selected six kinds of Chinese medicine herbals, which were highly correlated with liver cancer, and with no repetitive, effectiveness, hypotoxicity, and nonscarcity (Figure 1).

**Figure 2:**
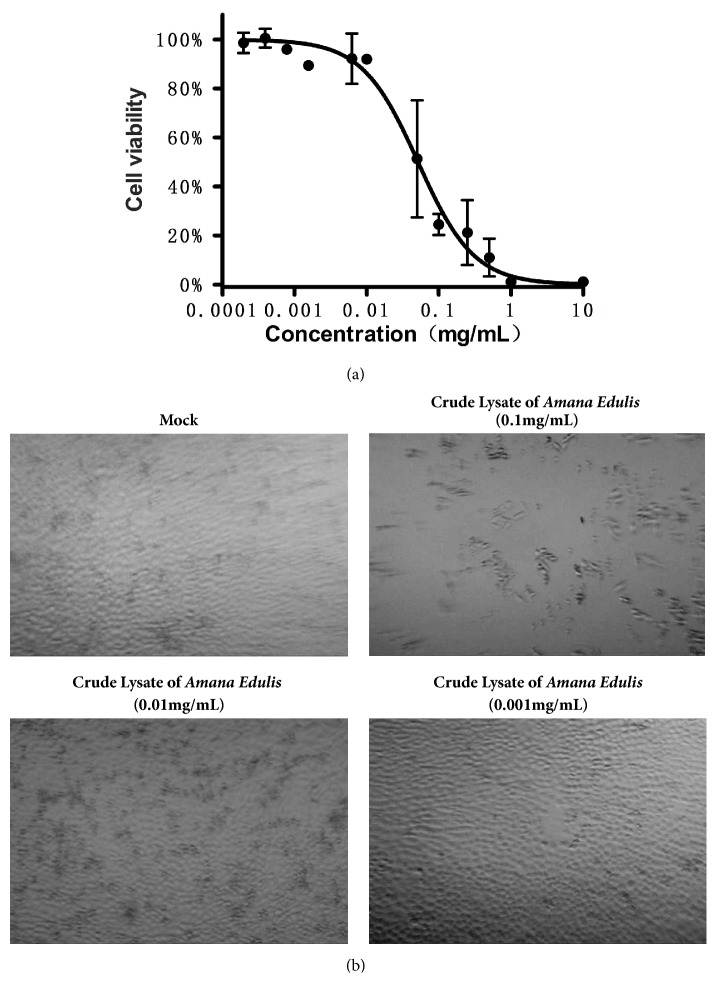
**The inhibition effects of crude lysate of* Amana edulis* in BEL7404 cells. (a) **BEL7404 cells were treated with the indicated doses of crude lysate of* Amana edulis* (concentration ranged from 0.0001mg/mL to 10mg/mL), and IC50 was calculated in BEL7404 cell lines.** (b)** BEL7404 cells were treated in the water-ethanol solvent of crude lysate of* Amana edulis* at various concentrations.

As shown in Table 1, two kinds of methods, water-ethanol and petroleum ether-ethyl acetate, were used to obtain crude extraction. The inhibitory effects of six ingredients were determined. All ingredients showed significant ability to inhibit the growth of BEL7404 at the concentration of 10mg/mL, whilst only* Prunella vulgaris L., Paeonia lactiflora Pall.,* and* Amana edulis* in water-ethanol solvent and* Prunella vulgaris L. and Vaccaria segetalis *(*Neck*.)* Garcke* in Petroleum Ether-ethyl acetate retained the ability at the concentration of 0.1mg/mL. We evaluated the effect of* Amana edulis* on the growth of the BEL7404 cells, and the data showed that IC50 of* Amana edulis* after being treated for 24h in BEL7404 cells was 0.052mg/mL (Figure 2(a)), which indicated that the crude extraction of* Amana edulis* is able to suppress the growth of BEL7404 cell lines. Moreover,* Amana edulis* shows the highest efficiency in the inhibition of the proliferation of liver cancer cells at the concentration of 0.1 mg/mL (Figure 2(b)).

### 3.2. The Crude Extraction of* Amana edulis* Induces DNA Fragmentation

**Figure 3:**
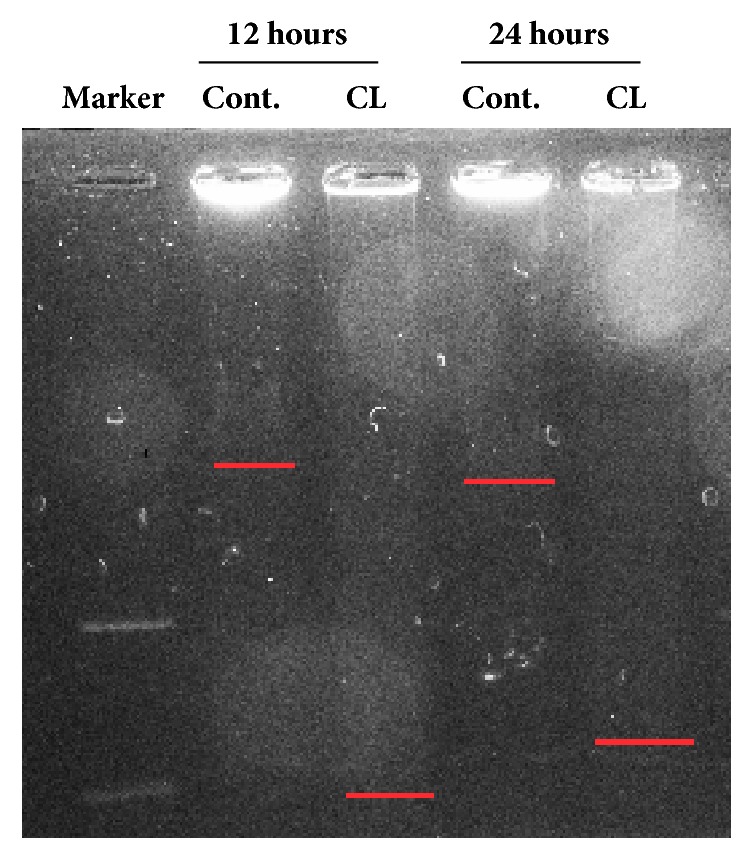
**Detecting the condition of DNA followed by the treatment of crude lysate of* Amana edulis*.** BEL7404 cells were treated with crude lysate of* Amana edulis *to check the condition of DNA by using DNA agarose gel electroless. Cells without any treatment were used as the baseline control.

To observe and measure the effect of* Amana edulis* on hepatoma carcinoma cell DNA, agarose gel electrophoresis had been implemented. The comet tails of the experiment group, which was treated with 0.1 mg/mL crude extraction of* Amana edulis*, was obviously longer than control group (Figure 3). Moreover, our results indicated that the DNA fragmentation may happen as early as 12h posttreatment, and* Amana edulis* may induce BEL7404 cells apoptosis.

### 3.3. The Crude Extraction of* Amana edulis* Induces Early and Late-Age Apoptosis of BEL7404 Cells

In order to explore the mechanism of apoptosis, Annexin V-FITC-PI Apoptosis Detection Kit was used to check whether apoptosis was induced.

**Figure 4:**
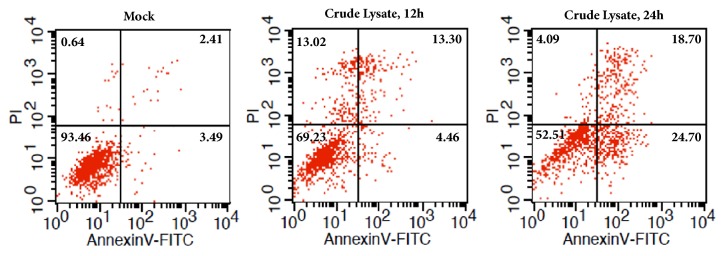
**Early and late-age apoptosis followed by the treatment of crude lysate of* Amana edulis*. **The BEL7404 cells were treated with the crude extraction of* Amana edulis* in water-ethanol solvent for various periods. Annexin V FITC-PI Apoptosis Detection Kit was used to check early-age and late-age apoptosis.

The flow cytometry data showed that early apoptosis values are 3.49% to 24.70% with beginning and ending difference of 7.08 times, and late apoptosis values are 2.41% to 18.70% with beginning to ending difference of 7.76 times (Figure 4). These results indicated that* Amana edulis* (water-ethanol) mixed system extract can significantly induce early and late-age apoptosis of BEL7404 cells.

### 3.4. The Crude Lysate of* Amana Edulis* Retards the Cell Reproduction at G2/M Stage

**Figure 5:**
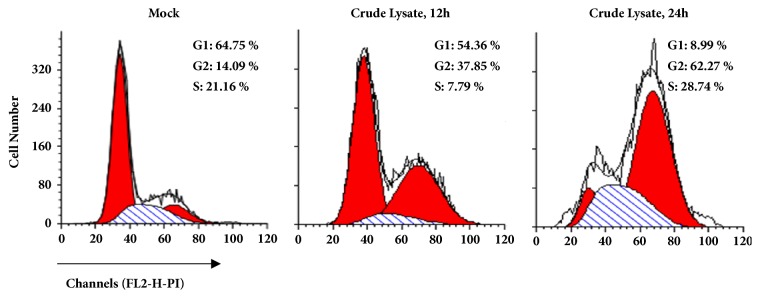
**Crude lysate of* Amana Edulis* induces cell cycle arrest at the G2/M phase. **BEL7404 cells were treated with crude lysate of* Amana edulis *for 12 and 24 hours. Cells were stained with propidium iodide, and the cell cycle distribution was analyzed by flow cytometry.

To investigate the inhibition mechanism of the crude lysate of* Amana Edulis* mediated cell cycle arrest at the G2/M phase, cell cycle assay was carried out.* Amana edulis *(water-ethanol) extract significantly arrested BEL7404 cells at G2/M phase after 24 hours' action, which may inhibit related protein activity during the division cycle (Figure 5).

### 3.5. The Crude Lysate of* Amana edulis* Has Inhibitory Effects on Other Liver Cancer Cell Lines

**Figure 6:**
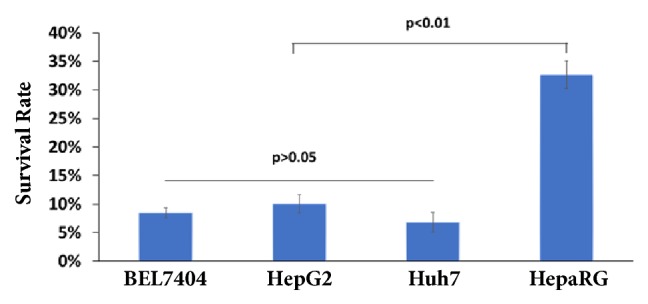
**The inhibitory effects on various cell lines.** BEL7404, HepG2, and Huh7 and HepaRG cells were treated with the crude lysate of* Amana edulis* at the concentration of 0.1mg/mL; MTT assay was conducted at 24h posttreatment.

To investigate whether this ingredient would have effect on other cell lines, human liver cancer cells BEL7404, HepG2, and Huh7 and normal liver cell HepaRG were used to conduct the verification experiment through MTT assay and t-test. The survival rate of HepaRG cell was the highest, followed by HepG2 cell, and the survival rate of Huh7 cell was the lowest (Figure 6). The results showed that the inhibition effect of* Amana edulis* (water-ethanol) mixed system extract on liver cancer cells have extremely significant difference compared with normal cells.

## 4. Discussion

Hepatocellular carcinoma (HCC) is a common malignant disease and is famous for its high incidence and mortality rates [23]. Moreover, at present, the therapeutic strategy available for the treatment of HCC is limited. A report showed that surgical intervention is currently one of the most effective methods for the treatment of HCC [24]. It is unfortunate that only a few patients with liver cancer are eligible for surgical treatment, because HCC is usually diagnosed at a late stage, due to the unresponsiveness of HCC cells. Therefore, systemic chemotherapy is still in the dominant position for the majority of patients, who is suffering from the injury of hepatocellular carcinoma [25]. However, It is well known that chemotherapy is doing harm to the body of the HCC patient and often accompanied with many side effects Therefore, it is a matter of great urgency to carry out research on hepatoma treatment, and new drug targets as well as chemical entities are in desperate need.

Chinese herbal medicine has been actively researched through various approaches. It has been increasingly accepted that Chinese herbal medicine plays an effective function in fighting against various diseases with low side effects, including HCC. In addition, it had been widely reported that some Chinese herbal medicines had an ability to inhibit the HCC at preclinical and clinical levels [12, 26].* Paeonia lactiflora Pall. root* had been proved to be a component of effective prescriptions for treatment of liver disease [7]. Astragalosides, the extractive of* Radix Astragali*, significantly induced apoptosis and inhibited invasion of tumor cells [27, 28].

Some Chinese herbal medicines in this study have been widely studied and even have been used in clinics. The extracts of* Prunella vulgaris L.* have multitarget and multipathway effects on antilung adenocarcinoma due to the regulation of steady state of calcium ion, cell cycle, and its steady state and the inhibition of tumor cell proliferation and metastasis [29]. The* Gnaphalium affine D. Don* is a folk medicine and used to treat antiinflammatory, and expectorant activities, and the extract of* Gnaphalium affine D. Don* showed significant antiinflammatory activity and reduced the paw swelling on MSU crystal-induced paw edema model [30]. Polysaccharides fractionated from roots of* Bupleurum chinense DC* present anticomplementary activity in vitro [31].* Vaccaria segetalis (Neck.) Garck*e [32] and* Paeonia lactiflor*a* Pall.* [33] were also extensively research.

*Amana edulis* has the effects of clearing heat, dissipating phlegm, and resolving masses with meridian distribution of liver and spleen, which is one of the anticancer Chinese medicinal herbs. In this study, our data indicated that six kinds of Chinese herbal medicines were certain antihepatoma effects, finding* Amana edulis* (water-ethanol) mixed system extract has the highest efficiency in the inhibition of the proliferation of liver cancer at the concentration of 0.1mg/mL. However, the effect of* Amana edulis* (water- ethanol) mixed system extract should be further investigated.

In this study, it was also observed that DNA showed a long tail in* Amana edulis* (water-ethanol) mixed system extract. These extracts can also increase early and late-age apoptosis rates and induce cell cycle arrest at the G2/M phase, indicating* Amana edulis *(water-ethanol) mixed system extract that may induce apoptosis. As is known to all, the proliferation and apoptosis of tumor cells are closely related to the regulation of cell cycle. For cancer cells, the blocking effect is an important index to evaluate the effect of antitumor therapy [34]. Cell cycle regulation is the key to cell proliferation [35]. Ophiopogonin D significantly inhibits cell proliferation and colony formation in MCF-7 breast cancer cells [36]. In ophiopogonin D-induced cell cycle was arrested at the G2/M phase in MCF-7 cells when the expression level of cyclin B1 was decreased [36]. In addition, MTT assay indicated that* Amana edulis *(water-ethanol) mixed system extract may be involved in the inhibition of the antitumor effect.

In conclusion, our findings indicated that* Amana edulis *(water-ethanol) mixed system extract contained potential high-activity anticancer effects on liver cancer cells in vitro. However, the effects of* Amana edulis* in vivo need to be further studied and further separation of single active component is expected to research its effect target. Moreover, more attention should be paid to consideration to develop the new drugs derived from* Amana edulis* for the treatment of liver cancer. In the struggle against HCC, we need not only the wisdom but the knowledge to solve the fatal problems that have plagued the whole world.

## Figures and Tables

**Table 1:** Comparison of the inhibitive effects of the twelve ingredients on liver cancer cell BEL7404.

Ingredients	Inhibition Rate, %			
	Water-Ethanol		Petroleum Ether-Ethyl Acetate	
	0.1 mg/mL	10 mg/mL	0.1 mg/mL	10 mg/mL
*Prunella vulgaris L.*	100%	100%	50%	90%
*Vaccaria segetalis (Neck.) Garcke*	0%	100%	90%	100%
*Gnaphalium affine D. Don*	0%	90%	0%	100%
*Amana edulis*	100%	100%	50%	100%
*Bupleurum chinense DC.*	0%	90%	0%	100%
*Paeonia lactiflora Pall.*	100%	100%	0%	90%

## Data Availability

The data used to support the findings of this study are available from the corresponding author upon request.
